# Long Non-Coding RNA LINC02747 Promotes the Proliferation of Clear Cell Renal Cell Carcinoma by Inhibiting miR-608 and Activating TFE3

**DOI:** 10.3389/fonc.2020.573789

**Published:** 2020-12-23

**Authors:** Xiang Ju, Yangyang Sun, Feng Zhang, Xiaohui Wei, Zhenguo Wang, Xiaozhou He

**Affiliations:** ^1^ Department of Urinary Surgery, The Third Affiliated Hospital of Soochow University, Changzhou, China; ^2^ Department of Gastrointestinal Surgery, The Third Affiliated Hospital of Soochow University, Changzhou, China

**Keywords:** ceRNA, ccRCC, miR-608, LINC02747, TFE3

## Abstract

With the rapid development of biotechnology, long noncoding RNAs (lncRNAs) have exhibited good application prospects in the treatment of cancer, and they may become new treatment targets for cancer. This study aimed to explore lncRNAs in clear cell renal cell carcinoma (ccRCC). Differentially expressed lncRNAs in 54 pairs of ccRCC tissues and para-carcinoma tissues were analyzed in The Cancer Genome Atlas (TCGA), and the most significant lncRNAs were selected and verified in ccRCC tissues. We found that lncRNA LINC02747 was highly expressed in ccRCC (*P* < 0.001) and was closely related to high TNM stage (*P* = 0.006) and histological grade (*P* = 0.004) and poor prognosis of patients (*P* < 0.001). In vivo and *in vitro* experiments confirmed that LINC02747 could promote the proliferation of ccRCC cells. We also found that LINC02747 regulated the proliferation of RCC cells by adsorbing miR-608. Subsequent mechanistic research showed that miR-608 is downregulated in ccRCC (*P* < 0.001), and overexpression of miR-608 inbibited the proliferation of RCC cells. Moreover, we found that TFE3 is a direct target gene of miR-608. MiR-608 regulated the proliferation of RCC cells by inhibiting TFE3. In conclusion, LINC02747 upregulates the expression of TFE3 by adsorbing miR-608, ultimately promoting the proliferation of ccRCC cells. The above findings indicate that LINC02747 acts as an oncogene in ccRCC and may be developed as a molecular marker for the diagnosis and prognosis of ccRCC. The LINC02747/miR-608/TFE3 pathway may become a new therapeutic target for ccRCC.

## Introduction

Renal cell carcinoma (RCC) is the most common malignant tumor originating from the kidney. According to statistics by Global Cancer 2018, RCC accounts for approximately 2–3% of all malignant tumors in adults (1). Recently, the morbidity and mortality rates of RCC have increased significantly, especially in young patients and those with high-grade tumors (2). According to the latest data from the Cancer Registry of the National Cancer Center of China, the incidence rate of RCC has also increased significantly in China year by year, and there were 45,096 new cases of RCC in 2011 (3). Clear cell RCC (ccRCC) is the most common and malignant subtype of RCC, accounting for 75–80% of RCC cases (4). Currently, radical nephrectomy is the major and most effective treatment modality for RCC. However, due to the lack of molecular markers for early diagnosis, approximately 25–30% of patients with RCC already show metastasis when diagnosed, with a median survival time of no more than 2 years (5). A total of 20–40% of patients with RCC suffer from metastasis after radical nephrectomy (6) and have a poor prognosis. Therefore, it is particularly important to clarify the molecular mechanisms underlying the development of ccRCC and identify molecular markers for early diagnosis and new therapeutic targets.

Approximately 70–90% of the human genome has transcriptional activity, but less than 2% of genes can encode proteins (7). With the completion of the human genome project and the improvement of deep sequencing techniques, 95% of transcripts have been shown to be noncoding RNAs (ncRNAs) (8). Long ncRNAs (lncRNAs) are longer than 200 nt in length. LncRNAs play an important role in various biological processes (9). LncRNAs have tissue-specific expression and play an important role in cell cycle regulation, cell growth, immune response, and cell pluripotency (9). More importantly, some lncRNAs are involved in multiple tumor-related pathways and play an important role in the occurrence and development of tumors (10). With the rapid development of biotechnology, lncRNAs have exhibited good application prospects in the treatment of cancer and may become new treatment targets for cancer. In this study, differentially expressed lncRNAs in ccRCC were analyzed in The Cancer Genome Atlas (TCGA), and the most significant lncRNAs were selected and verified in ccRCC tissues. We found that lncRNA LINC02747 was highly expressed in ccRCC tissues, indicating that LINC02747 may play an important role in ccRCC. There is currently no related research on LINC02747 in the literature. MiR-608 plays a role as a tumor suppressor gene in a variety of tumors. In this study, we found that miR-608 was expressed at low levels in ccRCC, and overexpression of miR-608 inhibited the proliferation of RCC cells. TFE3, a member of the MiTF family, is closely related to the occurrence and development of RCC and is the only driver gene inducing the formation of Xp11.2 (11, 12). Therefore, this study aims to explore the expression of LINC02747 in ccRCC cells and its effect on the proliferation of ccRCC cells, as well as the mechanism of LINC02747/miR-608/TFE3.

## Methods

### Ethical Statement

The study was approved by the Ethics Committee of the Third Affiliated Hospital of Suzhou University. All studies were conducted in accordance with the Helsinki Declaration. All patients in studies involving human subjects signed the informed consent forms. Experiments involving animal subjects were performed in accordance with the recommendations detailed in the Guide for the Care and Use of Laboratory Animals of the National Institutes of Health.

### Datasets

TCGA data were downloaded from The Atlas of ncRNAs in Cancer (TANRIC) software (https://ibl.mdanderson.org/tanric/_design/basic/main.html). A paired t-test was utilized to identify the differentially expressed lncRNAs between the 54 ccRCC and 54 paired paracarcinoma tissues. Differentially expressed lncRNAs with fold change ≥4 and adj *P*-value <0.01 were used for the subsequent analysis. The expression of LINC02747 in TCGA and related clinicopathological characteristics of 447 ccRCC patients were analyzed by t-tests. Kaplan–Meier curves were utilized to evaluate the prognostic value of LINC02747 in 447 ccRCC patients. Statistical analysis was performed with R software (version 3.5.3; https://www.r-project.org/) and SPSS 22.0 software (IBM, USA).

### Collection of ccRCC Tissue Specimens

Cancer and corresponding noncarcinoma tissues were collected from 20 patients undergoing radical nephrectomy at our hospital from June 2018 to December 2019. All patients were diagnosed for the first time and had not undergone any previous treatment. All patients were diagnosed with ccRCC *via* postoperative pathology, and all the tissue specimens were frozen in liquid nitrogen for later use.

### Cell Lines and Transfection

Cell lines (RPTEC/TERT1, 786-O, ACHN, Caki-1, and Caki-2) were purchased from American Type Culture Collection (ATCC, Rockville, MD, USA). Dulbecco’s modified eagle medium (DMEM) (Gibco, Carlsbad, CA, USA) with 10% fetal bovine serum (FBS) (Gibco, Carlsbad, CA, USA) was used for the cell culture. After the cells reached the logarithmic growth phase, the concentration was adjusted to 1×10^5^ cells/ml and then the cells were seeded into a 6-well plate containing slides for 24 h. Based on the manufacturer’s protocol for Lipofectamine 2000 (Invitrogen, Carlsbad, CA, USA), 75% confluent cells were transfected with 50 ng/ml of LINC02747 siRNA, sh-NC, sh-LINC02747, TFE3 specific siRNA, siRNA negative control, miR-NC, miR-608 mimics, miR-608 inhibitor, control, LINC02747, and TFE3 overexpression plasmid. All plasmids were purchased from BoyaBio Co. Ltd. (Shanghai, China).

### Luciferase Reporter Assay

The binding sites of LINC02747, TFE3, and miR-608 were predicted using a database. LINC02747-WT and TFE3-WT luciferase plasmids containing wild-type binding sites and LINC02747-MUT and TFE3-MUT containing mutated binding sites were inserted into the psiCHECK-2 luciferase reporter vector (Promega Corporation, Madison, WI, USA). The luciferase plasmids LINC02747-MUT, LINC02747-MUT, TFE3-WT, and TFE3-MUT were cotransfected with miR-608 mimic into 786-O and Caki-1 cells. After 48 h of culture, double luciferase system was used to detect the fluorescence value of each group.

### Quantitative Real-Time Polymerase Chain Reaction (qRT-PCR)

After transfection for 24 h, total RNA was extracted from cells with the manufacturer’s instructions (Invitrogen, Carlsbad, CA, USA). Then, the RNA was reverse transcribed into cDNA using the TaKaRa Reverse Transcription Kit (TaKaRa, Tokyo, Japan) under RNase-free conditions. The primers for the related genes and internal reference were designed and synthesized by Ximao Biotechnology Company (Shanghai, China), and the primer sequences are shown in **Table 1**. With GAPDH as an internal reference, the experimental results were obtained using the 2^-ΔΔCt^ method.

**Table 1:** Primers for qRT-PCR

Gene name	Forward primer (5’-3’)	Reverse primer (5’-3’)
LINC02747	CTGGGGTGATCCAAGGGTTC	AGACAAAGAGGCGTTCCCTG
BRD4	ACCTCCAACCCTAACAAGCC	TTTCCATAGTGTCTTGAGCACC
SSBP3	CCTTCTTATCGGAGATTCGATGG	ACACCACCACGAGTGCAAAAA
FOXO6	GTGGGGGAACCTTTCCTACG	TTCTGCACGCGGATGAACC
SOD3	ATGCTGGCGCTACTGTGTTC	CTCCGCCGAGTCAGAGTTG
TFE3	CCGTGTTCGTGCTGTTGGA	GCTCGTAGAAGCTGTCAGGAT

### Western Blot Analysis

Protein was extracted from 786-O and Caki-1 cells after transfection for 48 h. Then, the samples were transferred to a polyvinylidene fluoride (PVDF) membrane, followed by successive incubation with 5% nonfat milk, primary antibodies and secondary antibodies. The relative expression of the target protein was analyzed with GAPDH as a loading control. The primary antibodies anti-TFE3 (ab196681, 1:1,000, Abcam, UK) and anti-GAPDH (60004-1-Ig, 1:5,000, ProteinTech Group, USA) were used in this research. After incubation with secondary antibody, the protein bands were imaged using ImageJ software (Thermo Fisher Scientific, Waltham, USA). The experiment was performed three separate times.

### RNA Immunoprecipitation (RIP)

RIP analysis was performed using an RNA Immunoprecipitation Kit (Sigma-Aldrich Corporation, St. Louis, USA) in accordance with the manufacturer’s instructions, with an antibody against argonaute-2 (AGO2, Abcam, Cambridge, UK). IgG served as a control. The purified RNAs were then subjected to qRT-PCR analysis.

### Cell Counting Kit-8 (CCK-8) Proliferation Assay

Cells in the logarithmic growth phase were digested and transferred into a 96-well plate (approximately 2,000 cells/well). Cells were gently mixed evenly in the wells. After the cells adhered to the wall, they were transfected in each group for 0–96 h. After treatment, the culture solution was aspirated, and 90 μl of the corresponding complete medium and 10 μl of CCK-8 reagent (Dojindo, Kumamoto, Japan) were added for incubation in an incubator at 37°C for 1 h. Then, the absorbance value was measured at a wavelength of 450 nm, and the obtained data were statistically analyzed. The relative proliferation rate was calculated as the cell viability at 24, 48, or 72 h/cell viability at 0 h.

### Animal Experiments

The stably transfected cell lines in the control group in the logarithmic growth phase and the 786-O cell line stably transfected with control or sh-LINC02747 were digested with 0.25% trypsin, centrifuged, mixed with 1 ml of phosphate buffered saline (PBS), resuspended and counted. Then, the cells were prepared (approximately 1×10^7^ cells per nude mouse) and resuspended in 200 μl of PBS. The BALB/c nude mice (Soochow University, Jiangsu, China) were randomly divided into two groups: one group was injected with control cells, while the other group was injected with 786-O cells with LINC02747 knockdown. First, the axillary skin of the nude mice was disinfected with 1% iodine, and 200 μl of cell suspension was drawn into a disposable microsyringe and injected into the subcutaneous tissues of the axillary skin. Liquid leakage was carefully avoided and the grouping was labeled in detail. The nude mice were observed every 3 days to monitor the growth of the tumor and record the volume of the tumor. The tumor volumes were calculated as 0.5 × (length × width^2^). After 30 days, the nude mice were sacrificed *via* cervical dislocation, and the transplanted tumors were removed, weighed, imaged, and stored for subsequent tests.

### Statistical Analysis

SPSS 22.0 (IBM, USA) software was used for statistical analysis. The results are expressed as the mean ± standard deviation (mean ± SD). A *t*-test was used for comparison between two groups. One-way analysis of variance (ANOVA) with Tukey’s post-hoc test was used for the comparison between multiple groups, and data at different time points were processed by repeated-measures ANOVA, followed by a Bonferroni post-hoc test. The Kaplan-Meier method with the log-rank test was adopted for survival differences. A value of *P <*0.05 indicated significant difference.

## Results

### Identification of Differentially Expressed lncRNAs in ccRCC

**Figure 1:**
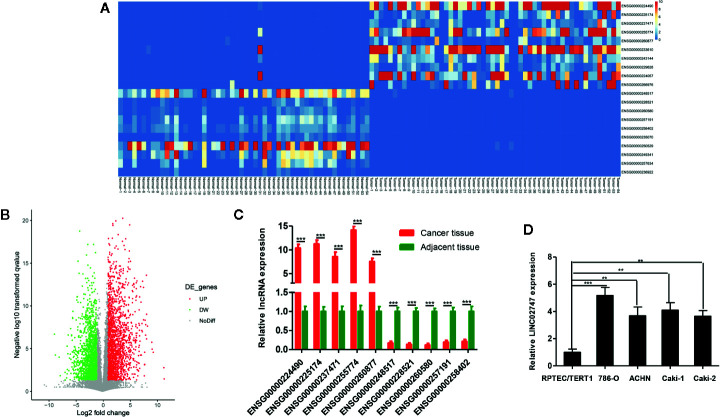
**(A)** Heatmap showing differentially expressed miRNAs based on 54 pairs of ccRCC tissues and matched para-carcinoma tissues in TCGA dataset (Fold Change ≥ 4 and *P* < 0.01). **(B)** Volcano plots were constructed based on TCGA (fold-change > 2.0 and P < 0.05). The red points represent differentially upregulated genes, and green points represent downregulated genes. **(C)** Validation of the lncRNAs in 20 pairs of cancer tissues and matched para-carcinoma tissues by qRT-PCR. **(D)** The expression of LINC02747 was detected by qRT-PCR in normal kidney cells (RPTEC/TERT1) and RCC cells (786-O, ACCN, Caki-1, and Caki-2). (^***^ *P* < 0.001, ^**^ *P* < 0.01, ^*^ *P* < 0.05).

The expression of lncRNAs was detected in a total of 54 pairs of ccRCC tissues and matched para-carcinoma tissues in TCGA database. With a fold change ≥4 and *p <*0.01 as the criteria, we found that a total of 20 lncRNAs were upregulated and 329 lncRNAs were downregulated in the ccRCC tissues (**Figures 1A, B**). The 5 most significantly upregulated lncRNAs in the ccRCC tissues (ENSG00000224490, ENSG00000225174, ENSG00000237471, ENSG00000255774, ENSG00000260877) and the 5 most significantly downregulated lncRNAs (ENSG00000248517, ENSG00000228521, ENSG00000260580, ENSG00000257191, ENSG00000258402) were selected and verified *via* qRT-PCR in 20 pairs of ccRCC tissues and para-carcinoma tissues. The results showed that the differential expression of ENSG00000255774 (LINC02747) was the most obvious. Compared with that in the para-carcinoma tissues, LINC02747 was highly expressed in the cancer tissues (*P* < 0.001) (**Figure 1C**). Then, the expression of LINC02747 was detected *via* qRT-PCR in normal kidney cells (RPTEC/TERT1) and RCC cells (786-O, ACCN, Caki-1, Caki-2). The results revealed that the expression of LINC02747 in the RCC cells was significantly higher than that in the normal kidney cells (**Figure 1D**). Therefore, LINC02747 was significantly highly expressed in the RCC cells and tissues.

### The Prognostic Value of LINC02747

**Figure 2:**
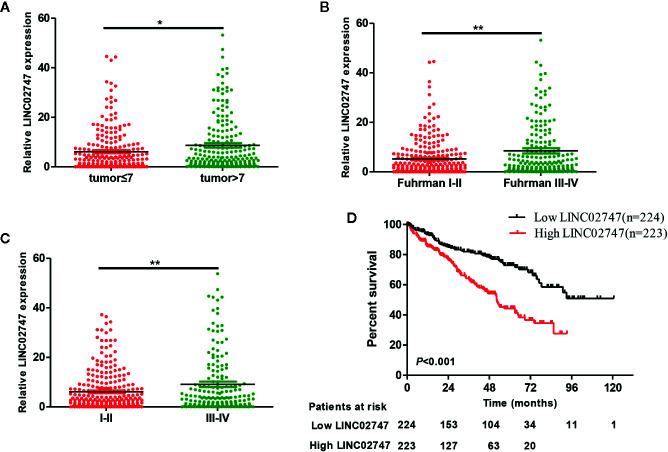
**(A)** The expression of LINC02747 in different tumor sizes based on TCGA dataset. **(B)** The expression of LINC02747 in different Fuhrman grades based on TCGA dataset. **(C)** The expression of LINC02747 in different TNM stages based on TCGA dataset. **(D)** The overall survival (OS) of patients with high LINC02747 expression was significantly less than that of patients with low LINC02747 expression. (^***^ *P* < 0.001, ^**^ *P* < 0.01, ^*^ *P* < 0.05).

The expression of lncRNAs in TCGA and related clinicopathological characteristics of 447 ccRCC patients were analyzed. The results showed that the expression of LINC02747 in the ccRCC patients with tumors >7 was significantly higher than that in the patients with tumors ≤7 (*P* = 0.019) (**Figure 2A**). The expression of LINC02747 was significantly higher in the patients with Fuhrman grade III–IV ccRCC than in those with Fuhrman grade I–II ccRCC (*P* = 0.004) (**Figure 2B**). The expression of LINC02747 was significantly higher in the patients with TNM stage III–IV ccRCC than TNM stage I–II ccRCC (*P* = 0.006) (**Figure 2C**). With the median value as the cut-off value, the overall survival (OS) of the patients with high LINC02747 expression was significantly lower than that of the patients with low LINC02747 expression [hazard ratio (HR) = 2.32, 95% confidence interval (CI) 1.66–3.24, *P* < 0.001] (**Figure 2D**).

### Effect of LINC02747 on the Proliferation of RCC Cells

**Figure 3:**
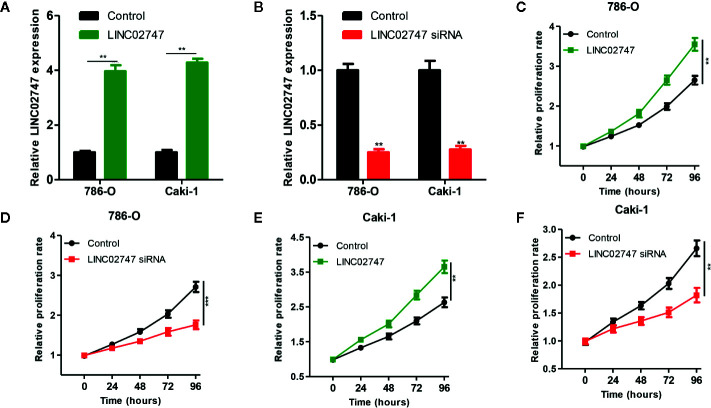
Verification efficiency of overexpressed LINC02747 **(A)** or downregulated LINC02747 **(B)** in 786-O and Caki-1 cell lines by qRT-PCR. After alteration of LINC02747 expression, the proliferation of 786-O **(C, D)** and Caki-1 **(E, F)** cells was changed. (^***^ *P* < 0.001, ^**^ *P* < 0.01, ^*^ *P* < 0.05).

To study the effect of LINC02747 on the proliferation of RCC cells, we overexpressed or silenced LINC02747 in 786-O and Caki-1 RCC cells by transfection of an overexpression plasmid or siRNA, respectively. The expression of LINC02747 after transfection is shown in **Figures 3A**, **B** (all *P* < 0.01). CCK-8 assay were performed to detect the effect of LINC02747 expression on the proliferation of 786-O cells. The upregulation of LINC02747 significantly promoted the proliferation of RCC cells (*P* < 0.004) (**Figure 3C**), while the downregulation of LINC02747 inhibited the proliferation of RCC cells (*P* < 0.001) (**Figure 3D**). The same results were obtained in Caki-1 cells (all *P* < 0.01) (**Figures 3E, F**).

Knocking Down LINC02747 Inhibited the Proliferation of RCC Cells in Nude Mice

**Figure 4:**
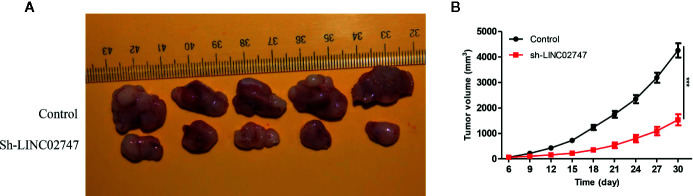
**(A)** Tumors collected from the control and sh-LINC02747groups for 30 days *in vivo*. **(B)** Growth curves of the Control and sh-LINC02747. (^***^ *P* < 0.001, ^**^ *P* < 0.01, ^*^ *P* < 0.05).

To further explore the effect of LINC02747 on tumor growth in nude mice, we injectd786-O cells with stably downregulated LINC02747 (sh-LINC02747) and control cells into the subcutaneous tissues of nude mice for 30 days. The growth rate of the tumors in the LINC02747 siRNA group was significantly lower than that in the control group (*P* < 0.01) (**Figures 4A, B**). The results showed that knocking down LINC02747 significantly inhibited the growth of RCC cells in nude mice, confirming the effect of LINC02747 on the growth of RCC cells *in vivo*.

### LINC02747 Regulated the Proliferation of RCC Cells by Inhibiting miR-608

**Figure 5:**
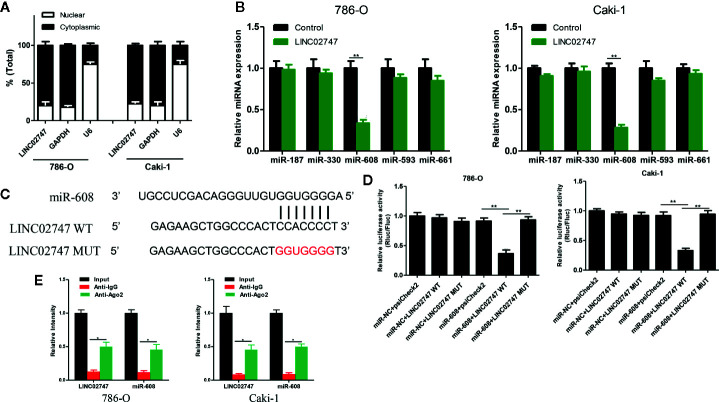
**(A)** qRT-PCR analysis of LINC02747 nuclear and cytoplasmic levels in 786-O and Caki-1 cells. U6 was used as a nuclear marker, and GAPDH was used as a cytosolic marker. **(B)** qRT-PCR detected the expression changes of the candidate miRNAs after overexpressing LINC02747 in 786-O and Caki-1 cells. **(C)** MiR-608 and LINC02747 binding sequences and LINC02747 mutated sequences. **(D)** A luciferase reporter assay was used to verify the direct binding between LINC02747 and miR-608. **(E)** The co-precipitated miR-608 was subjected to qRT-PCR for LINC02747 RIP experiments. (^***^ *P* < 0.001, ^**^ *P* < 0.01, ^*^ *P* < 0.05).

**Figure 6:**
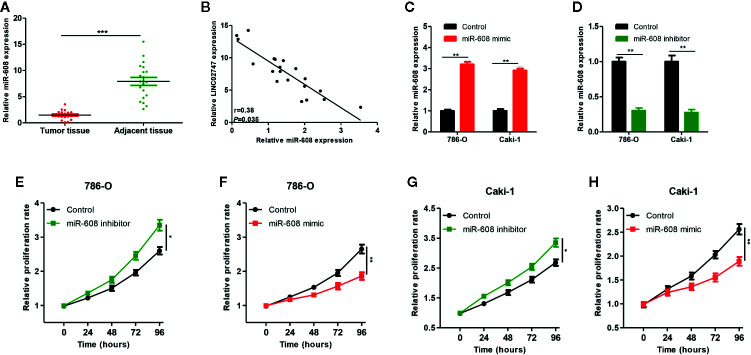
**(A)** qRT-PCR was used to detect miR-608 expression in 20 pairs of ccRCC tissues and para-carcinoma tissues. **(B)** The correlation between LINC02747 and miR-608 in 20 ccRCC tissues. Verification efficiency of overexpressed miR-608 **(C)** or downregulated miR-608 **(D)** in 786-O and Caki-1 cell lines by qRT-PCR. After alteration of miR-608 expression, the proliferation of 786-O **(E, F)** and Caki-1 **(G, H)** cells was changed. (^***^ *P* < 0.001, ^**^ *P* < 0.01, ^*^ *P* < 0.05).

Nuclear-cytoplasmic fractionation assay demonstrated that LINC02747 was mainly located in the cytoplasm (**Figure 5A**). Therefore, we hypothesized that LINC02747 may regulate the function of RCC cells by inhibiting miRNA. Using the RegRNA2.0 database (http://regrna2.mbc.nctu.edu.tw/), we identified the miRNAs (miR-187, miR-330, miR-608, miR-593, miR-661) most likely to bind to LINC02747. Then, LINC02747 was overexpressed in 786-O and Caki-1 cell lines, and the expression changes of the above miRNAs were detected by qRT-PCR. The results showed that miR-608 expression was significantly downregulated (all *P* < 0.01) (**Figure 5B**). Then, LINC02747-WT and LINC02747-MUT were cloned into the plasmid psiCHECK-2 (**Figure 5C**), and the constructs were cotransfected with miR-608 mimic into 786-O and Caki-1 cells for luciferase reporter assays. The results showed that miR-608 overexpression decreased the luciferase activity of the LINC02747-WT vector (*P* < 0.01) but did not reduce the luciferase activity of the mutant vector or empty vector (**Figure 5D**). RIP assays showed that LINC02747 and miR-608 were both contained in complexes that were pulled down with AGO2 antibody (**Figure 5E**). This result showed that LINC02747 can directly bind to miR-608, and the mutated sites are the binding sites between the two molecules. In addition, miR-608 expression was lower in cancer tissues than control tissues (*P* < 0.001) (**Figure 6A**), and the expression levels of LINC02747 and miR-608 were negatively correlated in 20 ccRCC tissues (r = 0.38, *P* < 0.001) (**Figure 6B**).

### Effect of miR-608 on the Proliferation of RCC Cells

To study the effect of miR-608 on the proliferation of RCC cells, we transfected miR-608 mimic and miR-608 inhibitor into 786-O and Caki-1 cell lines, and then the expression of miR-608 was detected using qRT-PCR. The expression of miR-608 after transfection is shown in **Figures 6C, D** (all *P* < 0.01). In the CCK-8 proliferation assay, we found that upregulation of miR-608 significantly promoted the proliferation of RCC cells (*P* = 0.023) (**Figure 6E**), while downregulation of miR-608 inhibited the proliferation of RCC cells (*P* < 0.01) (**Figure 6F**). The same results were obtained in Caki-1 cells (all *P* < 0.05) (**Figures 6G, H**).

### LINC02747 Regulated the Proliferation of RCC Cells by Inhibiting miR-608

**Figure 7:**
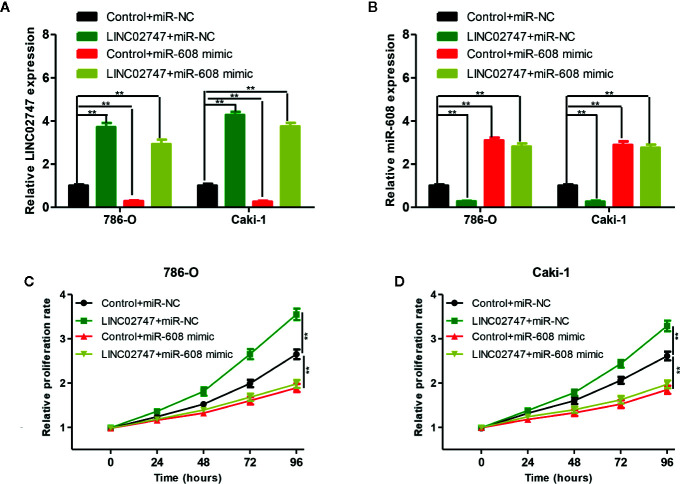
qRT-PCR analysis of LINC02747 **(A)** and miR-608 **(B)** expression in the786-O and Caki-1 cells transfected with Control+miR-NC, LINC02747+miR-NC, Control+miR-608 mimic, or LINC02747+miR-608 mimic. The 786-O **(C)** and Caki-1 **(D)** cells were transfected with Control+miR-NC, LINC02747+miR-NC, Control+miR-608 mimic, or LINC02747+miR-608 mimic., and cell proliferation in each group was detected by CCK-8 assays. (^***^ *P* < 0.001, ^**^ *P* < 0.01, ^*^ *P* < 0.05).

To explore whether LINC02747 regulates the proliferation of RCC cells by inhibiting miR-608, we transfected 786-O and Caki-1 cells with Control+miR-NC, LINC02747+miR-NC, Control+miR-608 mimic, or LINC02747+miR-608 mimic. The expression levels of LINC02747 and miR-608 in the four groups after transfection are shown in **Figures 7A, B** (all *P* < 0.01). CCK-8 proliferation assay showed that in the cell lines with upregulated miR-608, the upregulation of LINC02747 had no significant impact on the proliferation of RCC cells (**Figures 7C, D**). These results indicated that LINC02747 regulates the proliferation of RCC cells by inhibiting miR-608.

### Screening and Verification of miR-608 Target Genes

**Figure 8:**
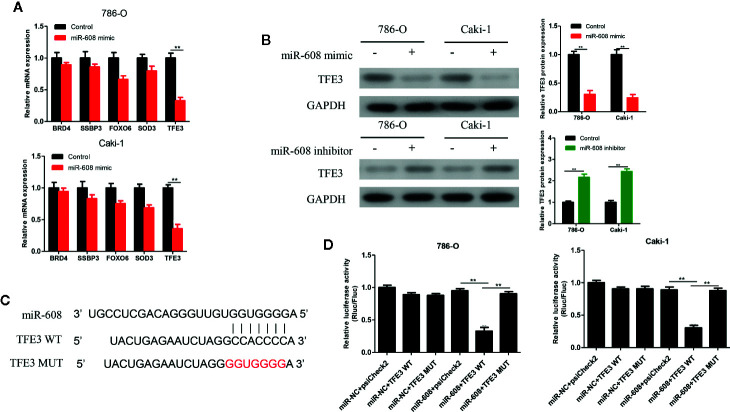
**(A)** qRT-PCR detected the expression changes of the candidate mRNAs after overexpressing miR-608 in 786-O and Caki-1 cells. **(B)** Western blot detected the TFE3 protein expression in the 786-O and Caki-1 cells transfected with miR-608 mimic or inhibitor. **(C)** MiR-608 and TFE3 binding sequences and TFE3 mutated sequences. **(D)** A luciferase reporter assay was used to verify the direct binding between TFE3 and miR-608. (^***^ *P* < 0.001, ^**^ *P* < 0.01, ^*^ *P* < 0.05).

The 5 most likely target genes of miR-608, namely, BRD4, SSBP3, FOXO6, SOD3, and TFE3, were selected as candidate target genes using TargetScan (http://www.targetscan.org/) and miRDB (http://mirdb.org/). Then, the expression of miR-608 in 786-O and Caki-1 cells was upregulated, and we found that the mRNA expression of TFE3 changed most significantly, and the difference was significant (*P* < 0.01, **Figure 8A**). Then, the expression of miR-608 was upregulated in 786-O and Caki-1 cells, and the TFE3 protein expression decreased, while the expression of TFE3 increased after downregulating miR-608 expression (all *P* < 0.01) (**Figure 8B**). Then, TFE3 wild-type and mutant sequences were cloned into the luciferase reporter plasmid psiCHECK-2 (**Figure 8C**), and the constructs were cotransfected with miR-608 mimic into 786-O and Caki-1 cells for the luciferase reporter assay. The results showed that overexpression of miR-608 could substantially decrease the luciferase activity of the wild-type TFE3 vector (all *P* < 0.01) but did not reduce that of the mutant or empty vector (**Figure 8D**). This result proved that TFE3 is the direct target gene of miR-608, and the mutated sites are the binding sites between the two molecules.

### MiR-608 Regulated the Proliferation of RCC Cells by Inhibiting TFE3

**Figure 9:**
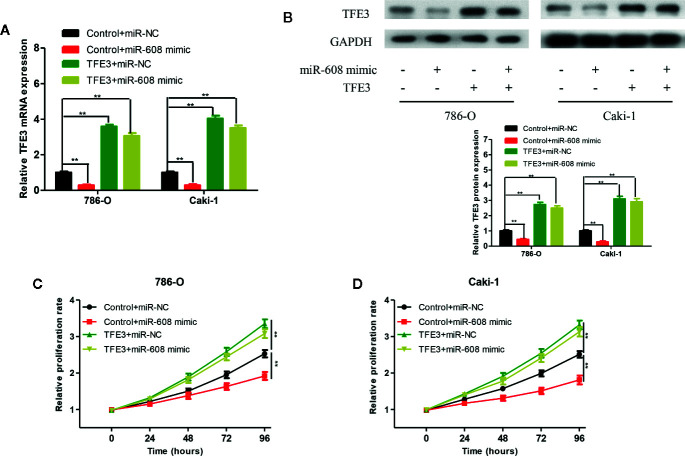
TFE3 mRNA **(A)** and protein **(B)** expression in the 786-O and Caki-1 cells transfected with Control+miR-NC, Control+miR-608 mimic, TFE3+miR-NC, or TFE3+miR-608 mimic. The 786-O **(C)** and Caki-1 **(D)** cells transfected with Control+miR-NC, Control+miR-608 mimic, TFE3+miR-NC, or TFE3+miR-608 mimic, and the cell proliferation in each group was detected by CCK-8. (^***^ *P* < 0.001, ^**^ *P* < 0.01, ^*^ *P* < 0.05).

To verify whether miR-608 can inhibit the proliferation of RCC by inhibiting TFE3, we transfected 786-O and Caki-1 cells with Control+miR-NC, Control+miR-608 mimic, TFE3+miR-NC, or TFE3+miR-608 mimics. The mRNA and protein levels of TFE3 in the four groups after transfection are shown in **Figures 9A** and **9B** (all *P* < 0.01). CCK-8 assays showed that upregulation of miR-608 expression had no significant impact on the proliferation of RCC cells when TFE3 was upregulated (**Figures 9C, D**). These findings showed that miR-608 regulates the proliferation of RCC by inhibiting TFE3. In summary, LINC02747 upregulates the expression of TFE3 by adsorbing miR-608, ultimately promoting the proliferation of RCC cells.

## Discussion

LncRNAs are the most important type of ncRNA in mammals (8), and they play an important role in various biological processes (9). In recent years, lncRNAs have become a research hotspot in cancer. The roles of lncRNAs in RCC and their biological function in the development of RCC have also attracted increasing attention from scholars. Xiao et al. found that lncRNA FILNC1 deficiency can reduce energy-induced apoptosis, thus significantly promoting the progression of RCC, and the mechanism involves increased glucose uptake of tumor cells and increased production of lactic acid through upregulation of c-Myc (13). Hong et al. reported that lncRNA HOTAIR competitively binds to miR-217, thereby upregulating its target gene HIF-1α and promoting the expression of downstream AXL genes, ultimately enhancing the proliferation, migration and epithelial-mesenchymal transition and inhibiting the apoptosis of RCC cells (14). According to another study, lncRNA DLX6-AS1 acts as an oncogene in RCC, and interference with DLX6-AS1 significantly inhibited the growth of RCC cells. Furthermore, DLX6-AS1 contains binding sites for miR-26a and promotes the progression of RCC by competitively binding to miR-26a and upregulating PTEN (15). Li et al. selected lncRNA MRCCAT1, which is closely related to ccRCC metastasis, through gene chip analysis. Overexpression of MRCCAT1 inhibited the transcription of NPR3, thereby activating the p38-MAPK signaling pathway and promoting the proliferation, migration, and invasion of RCC cells (16). In this study, lncRNAs related to ccRCC were screened in TCGA database and then verified using qRT-PCR. LncRNA LINC02747, which had the most significant difference in expression between cancer tissues and para-carcinoma tissues, was selected for further research. The *in vivo* and *in vitro* experiments confirmed that LINC02747 could promote the proliferation of RCC cells.

LncRNAs bind to miRNAs through complementary sequences and competitively downregulate the binding of miRNAs to target mRNAs, thus regulating gene expression, which is one of the main mechanisms of posttranscriptional regulation (17). In this study, LINC02747 was shown to regulate the proliferation of RCC cells by adsorbing miR-608. Multiple studies have reported that miR-608 plays a role as a tumor suppressor in various tumors. Wang et al. found that the expression level of miR-608 is significantly reduced in liver cancer tissues, and overexpression of miR-608 inhibited the proliferation of liver cancer cells through the G1 cell cycle (18). Yang et al. found that overexpression of miR-608 in colon cancer cell lines can remarkably inhibit the proliferation, cell cycle progression and migration of cancer cells (19). Wang et al. reported that overexpression of miR-608 in lung cancer cells can suppress the expression of TFAP4, thus promoting the apoptosis of NSCLC cells (20). Liang et al. found that the relative expression of miR-608 in bladder cancer tissues is low, and overexpression of miR-608 can cause cell cycle arrest and inhibit the proliferation of bladder cancer cell lines. Furthermore, miR-608 affected the downstream AKT/FOXO3a signaling pathway by targeting FLOT1, thus inhibiting the proliferation of bladder cancer (21). In this study, miR-608 was expressed at low levels in ccRCC, and overexpression of miR-608 inhibited the proliferation of RCC cells. Moreover, the results showed that TFE3 is a direct target gene of miR-608. MiR-608 regulated the proliferation of RCC cells by inhibiting TFE3. Studies have shown that TFE3 is a key pathway regulating cell development, with a wide range of physiological functions. TFE3, a member of the MiTF family, is closely related to the occurrence and development of RCC and is the only driver gene inducing the formation of Xp11.2 (11, 12). Studies have shown that TFE3 binds to Smad protein to activate the TGF-β signal transduction pathway and activate the PAI-1 gene (22). The TFE3 protein possesses potent transcriptional activity and can inhibit the p21-mediated pRB pathway, thereby causing uncontrolled cell proliferation and eventually leading to malignant transformation (23). Recently, it was reported that the occurrence of RCC is closely related to the inactivation of the tumor suppressor gene FLCN. After inactivation of FLCN, the TFE3 protein can be phosphorylated, and its aggregation in the nucleus is reduced, increasing TFE3 protein transcriptional activity and eventually upregulating the expression of hematopoietic stem cell growth factor-induced neuropeptide (HGFIN), thereby participating in the occurrence of RCC (24). Fang et al. found that overexpression of TFE3 can promote the proliferation and regulate the cycle of RCC cells mainly through excessive activation of the P13K/AKT/mTOR pathway (25). We look forward to future experiments to prove the ceRNA mechanism of LINC02747/miR-608/TFE3 in animal experiments and to verify the prognostic value of LINC02747 in ccRCC patient in large samples.

In conclusion, it was confirmed by analyzing the relevant data in TCGA and the qRT-PCR results of clinical specimens that lncRNA LINC02747 is highly expressed in ccRCC and is closely related to the high TNM stage and histological grade and the poor prognosis of patients. LINC02747 upregulates the expression of TFE3 by adsorbing miR-608, ultimately promoting the proliferation of RCC cells. The above findings indicate that LINC02747 acts as an oncogene in ccRCC and may be developed as a molecular marker for the diagnosis and prognosis of ccRCC. The LINC02747/miR-608/TFE3 pathway may become a new therapeutic target for ccRCC.

## Data Availability Statement

The original contributions presented in the study are included in the article/**Supplementary Material**. Further inquiries can be directed to the corresponding author.

## Ethics Statement

The studies involving human participants were reviewed and approved by Third Affiliated Hospital of Soochow University. The patients/participants provided their written informed consent to participate in this study. The animal study was reviewed and approved by Third Affiliated Hospital of Soochow University.

## Author Contributions

XJ and XH conceived and designed the study and helped to draft the manuscript. YS, FZ, and XW performed the data collection. ZW performed the statistical analysis. All authors contributed to the article and approved the submitted version.

## Conflict of Interest

The authors declare that the research was conducted in the absence of any commercial or financial relationships that could be construed as a potential conflict of interest.
