# Supplementary material for: Long Non-Coding RNA LINC02747 Promotes the Proliferation of Clear Cell Renal Cell Carcinoma by Inhibiting miR-608 and Activating TFE3
Source: Front Oncol. 2020 Dec 23;10:573789. doi: 10.3389/fonc.2020.573789 (PMC7786277; doi:10.3389/fonc.2020.573789)
Supplement: Supplementary file 1 [file Table_1.docx]

| Gene | Sequence of the primers |
| --- | --- |
| C5orf66-AS1-Forward | 5'-CGGGATCAACCCTCTGCTTT-3' |
| C5orf66-AS1-Reverse | 5'-TTCTTGAGAAGCGACTGCGT-3' |
| TFE3-Forward | 5'-CCCCTGCCATGTCGTCATC-3' |
| TFE3-Reverse | 5'-GTGGACGGCTCAATGTGTG-3' |
| BRD4-Forward | 5'- ACCTCCAACCCTAACAAGCC-3' |
| BRD4-Reverse | 5'-AGGTTGGACACCGTCTCATTC-3' |
| SSBP3-Forward | 5'-CCTTCTTATCGGAGATTCGATGG-3' |
| SSBP3-Reverse | 5'- ACACCACCACGAGTGCAAAAA-3' |
| FOXO6-Forward | 5'-CTGGGGTGATCCAAGGGTTC-3' |
| FOXO6-Reverse | 5'-AGACAAAGAGGCGTTCCCTG-3' |
| SOD3-Forward | 5'-ATGCTGGCGCTACTGTGTTC-3' |
| SOD3-Reverse | 5'-CTCCGCCGAGTCAGAGTTG-3' |
| GAPDH-Forward | 5'-GGAGCGAGATCCCTCCAAAAT-3' |
| GAPDH-Reverse | 5'-GGCTGTTGTCATACTTCTCATGG-3' |

Supplementary table 1 Primer sequences used for qPCR assays
